# Gelatin Methacryloyl Hydrogels Control the Localized Delivery of Albumin-Bound Paclitaxel

**DOI:** 10.3390/polym12020501

**Published:** 2020-02-24

**Authors:** Margaux Vigata, Christoph Meinert, Stephen Pahoff, Nathalie Bock, Dietmar W. Hutmacher

**Affiliations:** 1 Institute of Health and Biomedical Innovation (IHBI), Queensland University of Technology (QUT), Kelvin Grove, QLD 4059, Australia; m.vigata@qut.edu.au (M.V.); s.pahoff@qut.edu.au (S.P.); 2 School of Chemistry, Physics and Mechanical Engineering, Science and Engineering Faculty (SEF), Queensland University of Technology (QUT), Brisbane, QLD 4059, Australia; 3 Translational Research Institute, Woolloongabba, QLD 4102, Australia; 4 School of Biomedical Sciences, Faculty of Health, Queensland University of Technology (QUT), Brisbane, QLD 4059, Australia; 5 Australian Research Council Industrial Transformation Training Centre in Additive Biomanufacturing, Queensland University of Technology (QUT), Brisbane, QLD 4059, Australia

**Keywords:** drug delivery, breast cancer, gelatin methacryloyl hydrogel, Abraxane^®^, controlled release, localized adjuvant chemotherapy

## Abstract

Hydrogels are excellent candidates for the sustained local delivery of anticancer drugs, as they possess tunable physicochemical characteristics that enable to control drug release kinetics and potentially tackle the problem of systemic side effects in traditional chemotherapeutic delivery. Yet, current systems often involve complicated manufacturing or covalent bonding processes that are not compatible with regulatory or market reality. Here, we developed a novel gelatin methacryloyl (GelMA)-based drug delivery system (GelMA-DDS) for the sustained local delivery of paclitaxel-based Abraxane^®^, for the prevention of local breast cancer recurrence following mastectomy. GelMA-DDS readily encapsulated Abraxane^®^ with a maximum of 96% encapsulation efficiency. The mechanical properties of the hydrogel system were not affected by drug loading. Tuning of the physical properties, by varying GelMA concentration, allowed tailoring of GelMA-DDS mesh size, where decreasing the GelMA concentration provided overall more sustained cumulative release (significant differences between 5%, 10%, and 15%) with a maximum of 75% over three months of release, identified to be released by diffusion. Additionally, enzymatic degradation, which more readily mimics the in vivo situation, followed a near zero-order rate, with a total release of the cargo at various rates (2–14 h) depending on GelMA concentration. Finally, the results demonstrated that Abraxane^®^ delivery from the hydrogel system led to a dose-dependent reduction of viability, metabolic activity, and live-cell density of triple-negative breast cancer cells *in vitro*. The GelMA-DDS provides a novel and simple approach for the sustained local administration of anti-cancer drugs for breast cancer recurrence.

## 1. Introduction

Breast cancer is the most commonly diagnosed and the leading cause of cancer deaths in women worldwide [1]. In the USA alone, it was estimated that breast cancer incidence would represent 30% of all cancer diagnoses in women and 15% of cancer deaths in women for the year 2019 [2]. Breast cancer treatment commonly involves a combination of different approaches and techniques such as radiation therapy, chemotherapy, hormone therapy, targeted therapy, and/or surgery. Treatment schemes usually require surgical removal of the primary tumor. While the common practice has evolved to favor breast-conserving surgeries (lumpectomy), around 37% of breast cancer-related surgeries are complete mastectomies [3]. Adjuvant therapies, such as radiotherapy or chemotherapy, are then used after surgery to prevent cancer recurrence. More precisely, adjuvant chemotherapy is relevant in most breast cancer stages, subtypes, and often consist of systemic taxane and/or anthracycline drug administration [4,5,6].

Chemotherapeutic drugs, e.g., Paclitaxel, are often hydrophobic and hence require solvent-based formulations to be safely administered. However, the usage of the solvent causes severe toxicities such as anemia, bleeding events, neuropathy, infection, severe hypotension, dyspnea, or hypersensitivity reaction [7]. Fortunately, recent advances in drug formulations have enabled the development of the next generation of chemotherapeutics. Abraxane^®^ is a prime example of an FDA (Food and Drug Administration)-approved, next-generation taxane drug. This smart design utilizes human serum albumin (HSA) as a drug carrier for Paclitaxel [8]. Albumin-bound Paclitaxel molecules, (respective ratio 9:1), form 130 nm diameter particles, making the taxane drug water-soluble, therefore eliminating solvent-related toxicity. The other major advantage of Abraxane^®^ is that it leverages the inherent capability of HSA to accumulate in the area of the tumor cells by binding to SPARC (secreted protein, acidic and rich in cysteine) which is highly expressed and secreted by cancer cells [9,10]. This mechanism allows a 33% increase in drug accumulation at the tumor site and a shorter injection time for patients (30 min instead of 3 h for Taxol^®^) with reduced toxicity [8,11,12,13]. Nevertheless, Abraxane^®^ may still induce severe systemic side effects to the patient, including neutropenia, weakness, infection, muscle or joint pain, nausea, alopecia, among others [12,14].

Local drug delivery using polymeric matrices has been widely explored in the last decades as a strategy to limit systemic toxicity [15,16] and particularly applied to chemotherapeutics by locally delivering the drug directly to the target tissue [17]. Moreover, such drug delivery systems (DDS) can be customized to sustain drug release, which enhances therapeutic efficacy [18,19,20,21,22,23,24,25]. Micelles [26,27,28], micro/nanoparticles [29,30], and hydrogels [31,32], are examples of DDS previously investigated for local drug delivery. Hydrogels present several advantages for local drug delivery since they can protect labile drugs from degradation while providing spatial and temporal control over the release. Consequently, hydrogel-based DDS can serve as a platform for delivering a wide range of different molecules. The high tunability of physicochemical properties enables to control release kinetics through diffusion or degradation of the matrix or polymer-drug interactions [33].

The ultimate aim of this work is to implant a suitable hydrogel-based DDS in the surgical site after mastectomy or lumpectomy with enhanced control over release kinetics, but that still presents with manufacturing simplicity, essential for adoption in the clinic. For this purpose, we chose a well-established semi-synthetic hydrogel, gelatin methacryloyl (GelMA), which is characterized by low immunogenicity, biocompatibility, biodegradability, and tunability [34]. Gelatin, a hydrolyzed form of collagen type I and other extracellular matrix proteins, intrinsically harbors natural cell-binding motifs as well as matrix metalloproteinase (MMP) cleavage sites. The functionalization of gelatin with methacryloyl groups enables GelMA to be cured using photo- or chemical crosslinking, thus providing control and tailoring capabilities over the physicochemical properties of the hydrogel [35]. Three-dimensional (3D) gelatin or GelMA hydrogels have been investigated as scaffolds for tissue engineering [36,37] as well as drug delivery of growth factors [38,39,40], antitumor agents [41,42], siRNA [43,44], and antimicrobial agents [45]. Interactions between the hydrogel and molecules or biomolecules can be influenced by chemical modification, hydrophobic interactions, or physical properties of the hydrogel-based DDS [33]. The stiffness of GelMA-based hydrogels is widely tailored by GelMA synthesis parameters, such as the bloom strength and the degree of functionalization (DoF), as well as crosslinking parameters, which include photoinitiator concentration, curing time, and hydrogel concentration.

The hydrogel mesh size, which is inversely correlated with hydrogel stiffness, is the predominant controlling parameter for drug diffusion. An increase in mesh size increases the diffusion rate, whereas reduced mesh size can slow down the diffusion or entrap the drug in the matrix [33]. Miri et al. studied the impact of the DoF and curing conditions on hydrogel stiffness and reported a slower release rate of bovine serum albumin when the GelMA concentration was increased [46]. More precisely, it is the difference between mesh size and drug size that controls drug diffusion rates. In fact, another study varied the hydrogel concentration to tailor the physical properties of the DDS, thereby tailoring the release rate, but, most importantly, it showed that the relationship between mesh size and drug size played a critical role in diffusion and release rates [40]. Finally, the possibility to tailor the release of therapeutic agents from gelatin hydrogels by enzymatic degradation has been reported and is useful for in vivo applications [41].

In this manuscript, we propose to use GelMA as a DDS for the protein-anticancer drug formulation Abraxane^®^. Several studies have reported the use of GelMA hydrogel for local drug delivery, yet, to the best of our knowledge, there is no study reporting the local controlled delivery of Abraxane^®^ using GelMA, nor any polymeric hydrogel matrix. We hypothesized that Abraxane^®^-loaded GelMA hydrogels allow for tunability of the drug release kinetics via tailoring of the hydrogel physicochemical properties, enabling application as a suitable and easy-to-use DDS to induce breast cancer cell cytotoxicity. We first characterized the biomaterial and confirmed the DoF of GelMA. The GelMA-DDS was manufactured using different GelMA concentrations, i.e., 5%, 10%, and 15%, to vary the mechanical properties of the system. The GelMA-DDS was then characterized in terms of drug encapsulation efficiency, mechanical compressive properties as well as swelling properties. We investigated the effect of GelMA concentration on the release rate of Abraxane^®^ *in vitro*, in PBS, and enzymatically, via collagenase degradation. Finally, the cytotoxic potential of the GelMA-DDS was evaluated against MDA-MB-231 breast cancer cells grown in 3D configuration using viability, metabolic activity, and live-cell density as indicators.

## 2. Materials and Methods

### 2.1. GelMA Synthesis

Gelatin (porcine skin, Type A, gel strength 300; Sigma Aldrich, St. Louis, MO, USA) was dissolved in phosphate-buffered saline (PBS, pH 7.4; Invitrogen, Carlsbad, CA, USA) at 50 °C with agitation, for 10–60 min to reach a final concentration of 10% gelatin *w/v*. Next, methacrylic anhydride (MAA) was added to the mixture at 0.6 g of MAA per 1 g of dissolved gelatin. The methacryloyl functionalization reaction proceeded for 1 h, under vigorous stirring at 50 °C. The unreacted MAA and low molecular weight by-products were removed by dialysis using a 12-kDa molecular weight cut-off dialysis membrane, against a large volume of demineralized or ultrapure water (MilliQ, Merck Millipore, Burlington, MA, USA), changed daily. A final pH adjustment to 7.4 was performed using 1 M NaHCO_3_ before filter-sterilization (0.2 μm pore size). The macromere solution was then freeze-dried and stored at −20 °C [47].

### 2.2. Characterization of GelMA Functionalization

Methacryloyl functionalization of GelMA was confirmed using proton nuclear magnetic resonance (^1^H MNR) with a Bruker Avance 600 MHz spectrometer at room temperature, using 1% *w/v* macromere concentration in 90% *v/v* H_2_O/10% *v/v* D_2_O. To quantitatively assess the degree of functionalization (DoF), a 2,4,6-trinitrobenzene sulfonic acid (TNBS) assay was conducted. Briefly, 0.1 M Sodium bicarbonate (NaHCO_3_, pH 8.5) with 0.01% *w/v* TNBS solution was prepared. Gelatin and GelMA samples were separately dissolved, at 37 °C for 1 h, in 0.1 M NaHCO_3_ buffer at a 10 mg/mL concentration. Subsequently, samples were separately mixed with 0.1 M NaHCO_3_ buffer to obtain macromere concentrations of 500 µg/mL. Serial dilutions (1:2) were prepared to cover the range from 500 µg/mL to 0 µg/mL. Triplicates of 200 µL of both GelMA and gelatin dilutions were transferred into a 96-well plate, mixed with 100 µL of 0.01% *w/v* TNBS solution, and incubated for 2 h, protected from light. The absorbance was measured at 335 nm [48,49].

### 2.3. Drug Encapsulation and GelMA Crosslinking

GelMA was dissolved in PBS at 37 °C to obtain a 20% *w/v* precursor solution. The drug (Abraxane^®^, Specialised Therapeutics, Singapore) was encapsulated to achieve the final amount of 37.5 µg or 75 µg of Abraxane^®^ per hydrogel sample for Dose 1 or Dose 2, respectively. Abraxane^®^ was suspended in a solution of 5%, 10%, or 15% *w/v* GelMA containing 0.5 mg/mL solution of Irgacure 2959 (1-[4-(2-hydroxyethoxy)-phenyl]-2-hydroxy-2-methyl-1-propanone, BASF, Ludwigshafen, RLP, Germany). The mixture was pipetted into a custom polytetrafluoroethylene (PTFE) casting mold to form disc-shaped hydrogels of 5 mm diameter, 1.8 mm height, and approximate volume of 35 µL [47]. UV crosslinking at 365 nm light at an intensity of ~2.6 mW/cm^2^ in a CL-1000 crosslinker (UVP, Upland, CA, USA) was applied for 30 min. All % concentrations are *w/v* unless specified otherwise. Hydrogel samples were stored at 4 °C until use.

### 2.4. Effective Swelling

Hydrogel mass (*n* = 9) was determined immediately after crosslinking and compared to the weight of the gels after overnight swelling in PBS at 37 °C. The difference between the wet masses, before and after swelling, was expressed as a percentage.

### 2.5. Compressive Mechanical Properties

An unconfined compression test was performed to investigate the compressive moduli, failure stress, failure strain, and the toughness of the drug-loaded hydrogels versus the control groups (*n* = 9), using an Instron 5848 microtester with a 500 N load cell (Instron, Melbourne, VIC, Australia). The hydrogels were placed overnight in PBS at 37 °C, before the test. A displacement rate of 0.01 mm/s was applied using a non-porous aluminum indenter on the hydrogels, which were immersed in PBS at 37 °C. The compressive modulus was determined from the slope of the strain-stress curve between 10% to 15% strain [47].

### 2.6. Release Study

#### 2.6.1. Fluorescent Labeling of Abraxane^®^

Fluorescein-5-isothiocyanate (FITC, Sigma Aldrich, St Louis, MO, USA) 50× stock solution was prepared by dissolving the dye in dimethyl sulfoxide (DMSO) at 10 mg/mL. A suspension of Abraxane^®^ was prepared in 0.1M Sodium bicarbonate buffer (NaHCO_3_ pH 9) to a 10 mg/mL final concentration. FITC solution was added to the suspension for a final FITC concentration of 0.2 mg/mL. The labeling reaction was performed overnight, at room temperature, under agitation, and protected from light. The product was transferred into dialysis cellulose tubing (Sigma Aldrich, St Louis, MO, USA) with a typical molecular weight cut-off of 14,000 Da to remove unbound FITC. Dialysis was carried out for 7 days against ultra-purified water, protected from light, at room temperature, and the water was changed every day. The content of the dialysis membrane was then transferred into a falcon tube and freeze-dried (protected from light). The resulting freeze-dried FITC-labeled Abraxane^®^ was resuspended in PBS to prepare a 20% *w/v* stock solution.

#### 2.6.2. Encapsulation Efficiency

Hydrogels (*n* = 4) were degraded overnight in 500 µL of 28 units/mL collagenase II (Gibco™ Collagenase, Type II powder, Thermo Fischer Scientific, Waltham, MA, USA) in Hank’s Balanced Salt Solution (Gibco™ HBSS, calcium, magnesium, no phenol red, Thermo Fischer Scientific, Waltham, MA, USA). The degradation was carried out at 37 °C and 300 rpm on a thermomixer compact (Eppendorf, Hamburg, Germany). The amount of FITC-labeled Abraxane^®^ was quantified using a fluorescence plate reader (BMG PolarStar), from emission at 590 nm (excitation 544 nm). Results were compared to the control groups, i.e., suspended FITC-labeled Abraxane^®^ in degradation solution.

#### 2.6.3. Release Assay

Two doses of FITC-labeled Abraxane^®^ were investigated in this study, 37.5 µg (Dose 1) and 75 µg (Dose 2) per 35 µL hydrogel disk. The release was performed in 1 mL of PBS (pH 7.4) in low protein binding tubes (Eppendorf Protein LoBind Tubes, Hamburg, Germany) at 37 °C under agitation (75 rpm in an orbital incubator), protected from light. The release was quantified using a fluorescence plate reader (BMG PolarStar) at 485 nm excitation and 520 emission wavelengths. The experiment was repeated independently three times, and results were compared with control groups of the free drug at the corresponding doses.

#### 2.6.4. Mathematical Modeling

The Korsmeyer-Peppas equation *M_t_*/*M*_∞_ = *kt^n^* was used to model the release and describe the transport mechanism. *M*_t_/*M*_∞_ is the fractional release of drug in time *t* (*M*_t_ is the amount of drug released at time *t* and *M*_∞_ is the total amount of drug-loaded in the DDS), *k* is the constant characteristic of the drug-polymer system, and *n* is the release exponent characteristic of the release mechanism. In the case of a cylinder matrix such as the proposed drug delivery system, if *n* < 0.45, the mechanism of the release follows quasi-Fickian diffusion; if *n* = 0.45, it follows Fickian diffusion and the dominating release mechanism is the diffusion. This equation is, however, applicable only for the first 60% of the fractional release [50,51].

### 2.7. Abraxane^®^ Aggregation

Gels containing FITC-labeled Abraxane^®^ were placed in a petri dish to be imaged by a CSU X-1 Yokogawa spinning disc confocal (Nikon, Tokyo, Japan) at 488 nm and 10× magnification. Imaris software (Bitplane, South Windsor, CT, USA) was used to reconstruct the obtained z-stacks into 3D animations. Calibration of pixel intensity and target object size thresholds were performed to ensure the identification of the objects, i.e., drug aggregates with the maximum intensity. Upon 3D reconstruction, the software provided quantitative data such as intensity, size, location, geometry for each object. The average volume of FITC-labeled Abraxane^®^ aggregate was reported over time and normalized to Day 0.

### 2.8. Degradation Characterization

To mimic the gel digestion *ex vivo*, each gel was digested in 500 µmL of 28 units/mL collagenase (Gibco™ Collagenase, Type II powder, Thermo Fischer Scientific, USA) at 37 °C and 300 rpm on a thermomixer compact (Eppendorf, Hamburg, Germany). Every hour, the hydrogels were weighed. The ratio of digested mass to the original mass over time was used to represent the degradation rate.

### 2.9. Cell Culture

#### 2.9.1. Maintenance

MDA-MB-231 cells in passages 20–30 were cultured in Dulbecco’s Modified Eagle’s Medium (DMEM, 10313-021, Thermo Fischer Scientific, Waltham, MA, USA) containing 10% *v/v* Fetal Bovine Serum (FBS, Hyclone, Logan, UT, USA) and 1% penicillin/streptomycin (P/S; Thermo Fisher Scientific, VIC, Australia). Cells were incubated at 37 °C and 5% CO_2_ in a humidified cell culture incubator. Media was changed every 3 to 4 days, and cells were passaged at approximately 75% confluency.

#### 2.9.2. 3D Cell Culture

The cells were trypsinized, counted, and suspended at 1 × 10^6^ cells/mL in a solution of 10% *w/v* GelMA containing 0.5 mg/mL Irgacure2959. The mixture was transferred into Teflon molds to form disc-shaped hydrogels of 5 mm diameter and 1.8 mm height. UV crosslinking at 365 nm light at an intensity of ~2.6 mW/cm^2^ in a CL-1000 crosslinker (UVP, Upland, CA, USA) was applied for 10 min. The gels were then placed in 24-well plates with 1 mL of appropriate culture media, which was changed every 3 to 4 days. Gels were left in culture for 8 days to allow for cell proliferation and aggregate/spheroid formation.

##### Drug Delivery System Evaluation

Two doses of Abraxane^®^ were investigated in this study: Dose 1: 37.5 µg and Dose 2: 75 µg. On Day 0, cell-laden gels were moved to a new 24-well plate, and drug treatment was applied either as a free drug added to 1 mL of media, or released from the GelMA-based drug delivery system (DDS) in 1 mL of culture media. On day 3, 5, and 7, assays were performed, and the media was changed. Metabolic activity was assessed using the AlamarBlue^®^ assay; (*n* = 3), and cell viability and live-cell density were determined using a live/dead FDA/PI (Fluorescein Diacetate/Propidium Iodide) staining of 3D cultures (*n* = 3). After the assays, the media was replaced by fresh appropriate media (no more drug was added in the free drug control). The experiment was repeated 3 times at different times (biological replicates).

##### Metabolic Activity Assay

A volume of 500 μL of a 10% *v/v* AlamarBlue^®^ solution in the appropriate media was used to incubate the samples for 4 h at 37 °C and 5% CO_2_ in a humidified cell culture incubator. Fluorescent emission at 590 nm (excitation 544 nm) was measured from a fluorescence plate reader (BMG PolarStar). Results were normalized to the control group (% control).

##### Cell Viability Assay

Cells were stained with Fluorescein Diacetate (FDA) and Propidium Iodide (PI). The hydrogels were washed in PBS then incubated in 10 μg/mL FDA and 5 μg/mL PI in PBS for 7 min at 37 °C and 5% CO_2_ in a humidified cell culture incubator then washed again. Z-stack images were taken with a Leica SP5 confocal laser scanning microscope (Leica, Wetzlar, Germany) at 10× magnification. Cell viability was calculated using the maximum projection of the z-stacks and expressed as a percentage of live cells to total cells.

##### Live Cell Density

The Imaris software (Bitplane, South Windsor, CT, USA) was used to reconstruct into 3D animations the live/dead *z*-stacks from the cell viability assay to determine the live cell density of cell-laden gels. Calibration of pixel intensity threshold and the target object size parameter were set to ensure the identification of the objects, i.e., live and dead cells with the maximum intensity. Upon 3D reconstruction, the software provided the total volume of z-stack analyzed as well as quantitative data such as intensity, size, location, and geometry for each object. The total number of live cells was divided by the *z*-stack volume of hydrogel construct analyzed to obtain the live cell density expressed in a number of cells per µm^3^ of hydrogel construct.

### 2.10. Statistical Analysis

Statistical analysis was performed using GraphPad Prism software (version 8, GraphPad, CA, USA). Probabilities of *p* ≤ 0.05 were considered a significant difference. Two-way ANOVA (analysis of variance) with Tukey’s post hoc was used to investigate the mean difference between groups for the physical and swelling properties of the drug delivery system. The significant differences in means between groups were determined using one-way ANOVA with Tukey post-hoc for the encapsulation efficiency, the average size of FITC labeled Abraxane^®^, and for the in vitro cell evaluation of the drug delivery system. The significance of mean differences between groups of in vitro release results was calculated using the general linear model (univariate analysis), using IBM SPSS Statistics 23 (IBM Corp).

## 3. Results and Discussion

This work aimed to assess GelMA hydrogels as a suitable carrier for the anticancer agent Abraxane^®^ for the local and controlled delivery into a mastectomy or lumpectomy surgery site to prevent local breast cancer recurrence. In this first section, we confirmed the successful methacrylation of gelatin and determined the DoF of GelMA. In the second section, we assessed whether the drug loading had an impact on the mechanical and swelling properties of the GelMA-DDS. Then, the encapsulation efficiency of the drug in the GelMA-DDS was measured. In section four, we describe and characterize the release kinetics in PBS of Abraxane^®^ Dose 1 and Dose 2 from the GelMA-DDS for various concentrations of GelMA (5%, 10%, and 15%), and in the fifth section, we evaluated the release of the same GelMA-DDS groups via enzymatic degradation. Finally, in the last section, we evaluated the in vitro cytotoxic effect of the GelMA-DDS against 3D breast cancer cells MDA-MB-231 and compared it with Dose 1 and Dose 2 suspended in the media, which are simulating an intravenous injection of the drug.

### 3.1. Material Characterization

**Figure 1:**
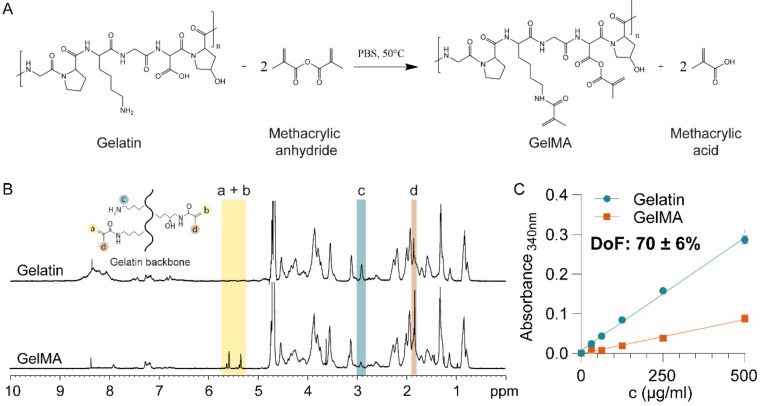
Gelatin Methacryloyl synthesis and characterization. (**A**) Gelatin Methacryloyl (GelMA) synthesis by methacrylation at 50 °C produces GelMA and methacrylic acid. (**B**) Proton nuclear magnetic resonance (^1^H-MNR) spectra of Gelatin and GelMA, confirming the substitution of primary amine groups by methacryloyl groups in GelMA. (**C**) Degree of GelMA functionalization, as measured by 2,4,6-Trinitrobenzene Sulfonic Acid (TNBS) assay.

First, GelMA was synthesized by the reaction of gelatin with methacrylic anhydride (Figure 1A) and characterized using proton nuclear magnetic resonance (^1^H NMR) (Figure 1B). The GelMA spectrum displayed peaks corresponding to acrylic protons of the methacryloyl grafts of lysine and hydroxyl lysine groups between 5–6 ppm (Figure 1B, peaks a + b), which were absent in the spectrum of gelatin. Peak c, at 2.9 ppm, was substantially smaller in the spectrum of GelMA, indicating that a large proportion of lysine groups were functionalized. Finally, peak d, corresponding to methyl protons of methacryloyl grafts (1.8 ppm), was present in the spectrum of GelMA, but not gelatin. Taken together, the ^1^H-NMR spectra confirmed successful methacryloyl functionalization of gelatin [52].

The DoF of GelMA influences the crosslinking density and, consequently, the mechanical properties of the hydrogel [47]. For drug delivery applications, the mechanical properties, particularly the mesh or pore size of the hydrogel, are the predominant drug release regulators [33]. We, therefore, quantitatively assessed the DoF using a 2,4,6-Trinitrobenzene Sulfonic Acid (TNBS) assay, which indicated that 70 ± 6% of lysine and hydroxy-lysine groups were functionalized (Figure 1C).

The DoF determines the maximal crosslinking density achievable, which impacts the mesh size [53,54] and thus the stiffness of the hydrogel [53,55]. When fully crosslinked, a hydrogel with a high DoF will display high stiffness and smaller mesh size. Furthermore, a higher stiffness means that the enzymatic degradation rate by metalloproteinase decreases, which is critical for in vivo applications [46]. An increase in GelMA concentration decreases the mesh size and therefore increases the hydrogel stiffness [47]. Increasing photoinitiator concentration increases the crosslinking kinetic rate so that maximal crosslinking can be achieved faster. Increasing curing time increases the crosslinking density therefore the stiffness and decrease the mesh size. Both curing time and photoinitiator concentration parameters are limited by the DoF [47]. Thus, achieving a fixed high DoF was essential as it allowed to obtain hydrogels with a wide range of achievable mesh sizes and stiffness, through the modification of concentration and crosslinking parameters, thus allowing the tailoring of drug release kinetics efficiently. Other studies indeed reported the use of a GelMA with high DoF ranging from around 70% to 87% for drug delivery applications [39,45].

### 3.2. Mechanical Properties of the Drug Delivery System

Following the successful manufacture of the GelMA-DDS, we determined whether drug loading had an impact on the photocrosslinking reaction and, therefore, the mechanical properties of the hydrogel. Based on the findings of an initial dose-effect study (Figure S1), we chose to encapsulate two doses of Abraxane^®^, 37.5 µg (Dose 1) and 75 µg (Dose 2), per 35 µL hydrogel sample, representing 1.07 µg/µL and 2.65 µg/µL of Abraxane^®^ per microliter of hydrogel. We encapsulated the drug in 5%, 10% and 15% GelMA, respectively, to cover a theoretical stiffness range from 5 kPa to 100 kPa [56] which may be useful in a wide range of applications and target tissues, and investigated the effects of modulating stiffness on release kinetics.

It is well-established that GelMA exhibits tunable swelling and mechanical properties. The determinant parameters are the DoF, the macromere concentration, and the curing parameters (photoinitiator concentration and curing time) [35]. Implantable hydrogels must be mechanically compliant with the surrounding environment or tissue of implantation and exhibit suitable degradation rate depending on the application. Moreover, the mechanical properties of hydrogels also impact the controlled release of drugs [57]. Therefore, we hypothesized that tailoring the mechanical properties of GelMA would allow the control and tailoring of the Abraxane^®^ release kinetics of the GelMA-DDS.

**Figure 2:**
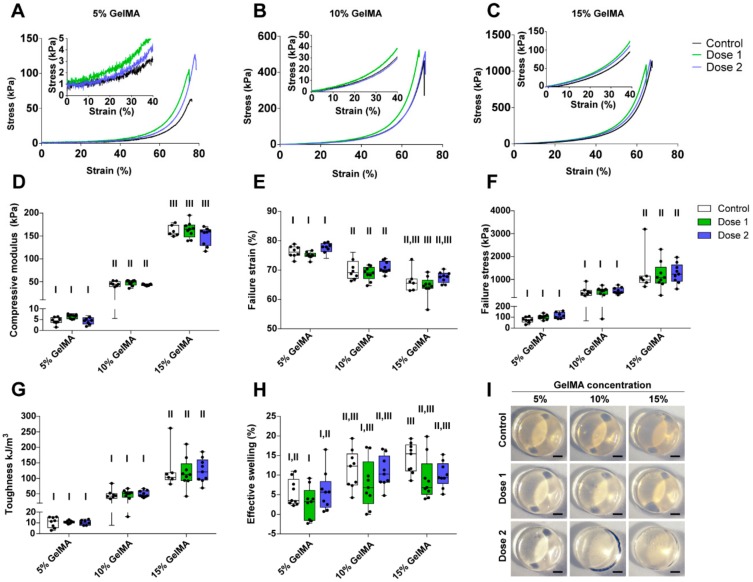
Characterization of the compressive and swelling properties of GelMA-DDS hydrogels for various GelMA concentrations and doses of Abraxane^®^. (**A**–**C**) Representative stress-strain curves for 5% *w/v* (**A**), 10% *w/v* (**B**) and 15% *w/v* (**C**) GelMA groups. (**D**) Compressive modulus. (**E**) Failure strain. (**F**) Failure stress. (**G**) Toughness. (**H**) Effective swelling. (**I**) Representative images of hydrogel samples; scale bar = 1 mm. Dose 1 and 2 refer to 37.5 µg and 75 µg Abraxane^®^, respectively. Control refers to no drug loading. Data are shown as box plots, *n* = 9. Groups with no statistical difference are marked with the same roman numeral.

Figure 2A–C show representative stress-strain curves of all groups. We first observed that the compressive modulus increased significantly with the increase of GelMA concentration and varied between 5 kPa to 150 kPa, depending on the GelMA concentration (Figure 2D). As the stiffness of the hydrogels increased, there was a significant increase in both failure stress and toughness, respectively, ranging from around 80 kPa to 1200 kPa and 10 kJ/m^3^ to 120 kJ/m^3^ (Figure 2F,G). In contrast, the failure strain decreased from approximately 75% to 65% with increasing GelMA concentration (Figure 2E). Hydrogel swelling tended to increase with GelMA concentration, but overall was not a dominating feature of the proposed GelMA-DDS (Figure 2H). All hydrogels presented with similar morphologies (Figure 2I). The most important observation was that within each GelMA concentration tested, there was no statistical difference between groups containing Abraxane^®^ and controls. This result proves that the loading of Abraxane^®^ did not alter the mechanical compressive properties of the GelMA-DDS for the hydrogel and drug concentrations tested.

### 3.3. Encapsulation Efficiency

Next, we investigated the encapsulation efficiency (EE), i.e., the percentage of Abraxane^®^ successfully encapsulated in the GelMA-DDS as compared to the initial amount of drug available for encapsulation, using FITC-labeled Abraxane^®^ (Table 1).

Hydrogels with high water content are a very favorable environments for protein drugs as they usually display high EE [33]. We observed no significant differences between the groups tested but noted that the average EE was lower for Dose 2 compared to Dose 1 for all GelMA concentrations. On average, the EE of Abraxane^®^ was lower in 15% GelMA, i.e., below 80%, whereas 5% and 10% GelMA achieved EE above 90%, which is high when compared to other studies [39,58,59,60,61,62]. To explain the loss of drug following encapsulation, we explored the potential effect of the UV exposure used for crosslinking of the GelMA-DDS on the fluorescence intensity of FITC-labeled Abraxane^®^. We found no statistical difference for FITC-labeled Abraxane^®^ before and after UV exposure for concentrations between 18.75 µg/mL to 75 µg/mL and, therefore, ruled out this possible cause (Figure S2). We then hypothesized that part of the drug remained attached to the Teflon casting mold used for the GelMA-DDS crosslinking. If the drug loss was due to surface attachment on the mold, we can infer that with a higher drug density in the same hydrogel volume, i.e., a higher dose, more drug particles would be lost at the hydrogel/mold interface. This would explain the lower EE for the Dose 2 compared to Dose 1 GelMA-DDS formulations.

### 3.4. Release Profile

We employed different GelMA concentrations to encapsulate Abraxane^®^ with the hypothesis that release would be more sustained with increasing macromere content due to reduced mesh size. To measure the release of Abraxane^®^ using fluorescence spectroscopy, we labeled the human serum albumin (HSA) part of the drug because it constitutes the majority of Abraxane^®^ (90%/10% HSA-Paclitaxel ratio). The fluorescent probe FITC is commonly used to label molecules of interests and especially albumin, which has been widely used as a model for protein delivery and is recognized as representative of the release profile of unlabeled compounds [63,64,65,66,67,68]. We assumed that the Paclitaxel-HSA bond remained stable throughout the assays since the detachment of Paclitaxel from HSA is triggered by the competition with other highly hydrophobic compounds such as fatty acids of the cell membrane [9,10,69], not present in the PBS in vitro system.

**Figure 3:**
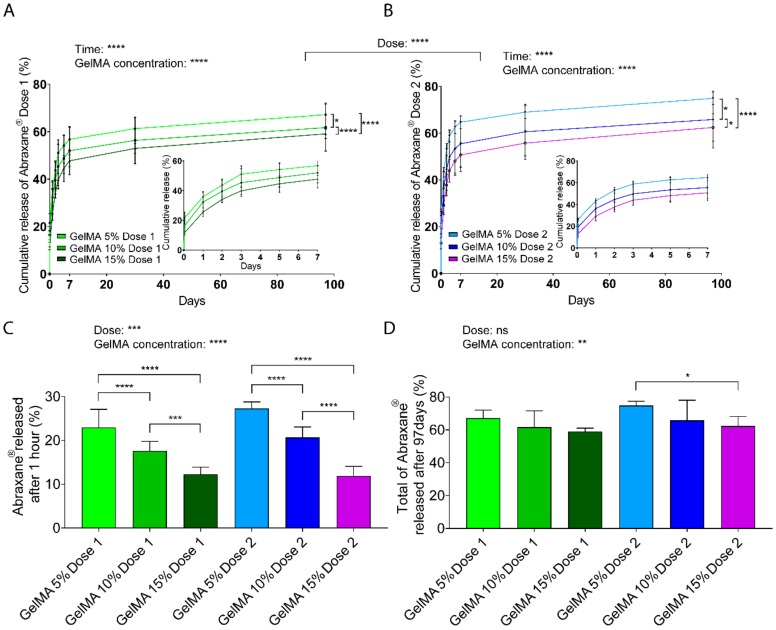
Release kinetics of FITC-labeled Abraxane^®^ from GelMA hydrogels, as measured in phosphate-buffered saline pH 7.4, under gentle agitation at 37 °C. (**A**–**B**) Cumulative release profiles, normalized to encapsulated Dose 1 (37.5 μg) (**A**) and Dose 2 (75 μg) (B) from 5%, 10%, and 15% w/v GelMA hydrogels. Data are shown as mean ± standard deviation (SD). (**C**–**D**) Fraction of FITC-Abraxane^®^ released in the first hour (**C**), and released after 97 days (**D**), normalized to GelMA encapsulated doses. Data are shown as mean + SD. GelMA Dose 1 and 2 refer to 37.5 µg and 75 µg Abraxane^®^, respectively. Data are shown as mean + SD. Release experiments were repeated three times, n ≥ 5. **** *p* < 0.0001; *** *p* < 0.001; ** *p* < 0.01; * *p* < 0.05.

Assessment of in vitro release (Figure 3A,B) suggested an initial burst release after the first hour (less than 30% of the total cargo), followed by sustained release of the drug for both doses and all GelMA concentrations. The burst release may partly stem from loosely attached drug particles at the surface of the GelMA-DDS, which are highly prone to diffusion. The effect of the hydrogel concentration was clearly demonstrated as, for both Abraxane^®^ doses, the amount of drug released significantly increased with decreasing GelMA concentrations. In other words, 15% GelMA displayed more sustained release than 10% GelMA, which, in turn, showed more sustained release than 5% GelMA. The overall cumulative release curves for Dose 1 formulations were also significantly different (*p* < 0.0001) compared to Dose 2 formulations. Maximum amounts released reached 67% and 75% from Dose 1 and Dose 2 formulations, respectively, from 5% GelMA gels. Since hydrogel mesh size decreases with increasing hydrogel concentration, the results suggest that with increasing GelMA concentrations, the mesh size decreased and slowed down drug diffusion or entrapped part of the drug, therefore inducing a more sustained release.

The release rate confirmed the burst release obtained within the first hour of the release with values above 10%/h for all groups and then decreased below 1%/h similarly for all groups and followed negative power functions (Figure S3A,B). The release mechanism was investigated using the mathematical model Korsmeyer-Peppas, which fitted the release data adequately with R^2^ values above 0.9237 (Figure S3C,D). The release exponent, *n*, of all tested conditions was below 0.45, indicating that the primary release mechanism was pseudo-Fickian diffusion (Table 2).

Focusing on the largest release timepoint, which corresponds to the first hour, there was a distinct and significant effect for both dose and GelMA concentration parameters on the amount of Abraxane^®^ released (Figure 3C). Dose 2 formulation displayed a higher released amount than Dose 1 formulation, and these values were reduced by increasing GelMA concentration in the GelMA-DDS. The absolute values (micrograms) released per timepoint are found in Figure S4A–C. Absolute values ranging from 4.5 to 20 µg were released after 1 h from the 35 µL hydrogel, according to different GelMA-DDS formulations, and followed an inverse linear correlation with GelMA concentration (*R*^2^ > 0.99), with a steeper slope for the higher dose formulation (Figure S4D). In summary, the highest release amount (first hour) can be strongly controlled by both the dose and GelMA concentration parameters, for the range of parameters tested.

At the end of the release assay (Day 97), the cumulative fractions released from GelMA-DDS formulations for both doses were significantly lower when the GelMA concentration was higher (Figure 3D). The GelMA-DDS formulations released from 59% to 67% (22.2 to 25.2 µg) and from 62% to 75% (46.5 to 56.1 µg) of Dose 1 and Dose 2, respectively, yet those differences were less significantly different than earlier time points, with no significant differences between different doses in GelMA-DDS formulations.

In summary, these results show that there is an apparent and expected overall effect of the GelMA concentration that significantly reduces the amounts of released drugs, thus increasing the sustainability of the release. Moreover, the fact that the fraction of Abraxane^®^ released for both doses was not statistically different for the same GelMA concentration confirms that GelMA concentration is a more dominant factor for drug release. In other words, to control the total amount of Abraxane^®^ released from GelMA, tailoring the hydrogel concentration is more important than the drug loading, for the range tested in this study.

### 3.5. Release through Enzymatic Degradation

**Figure 4:**
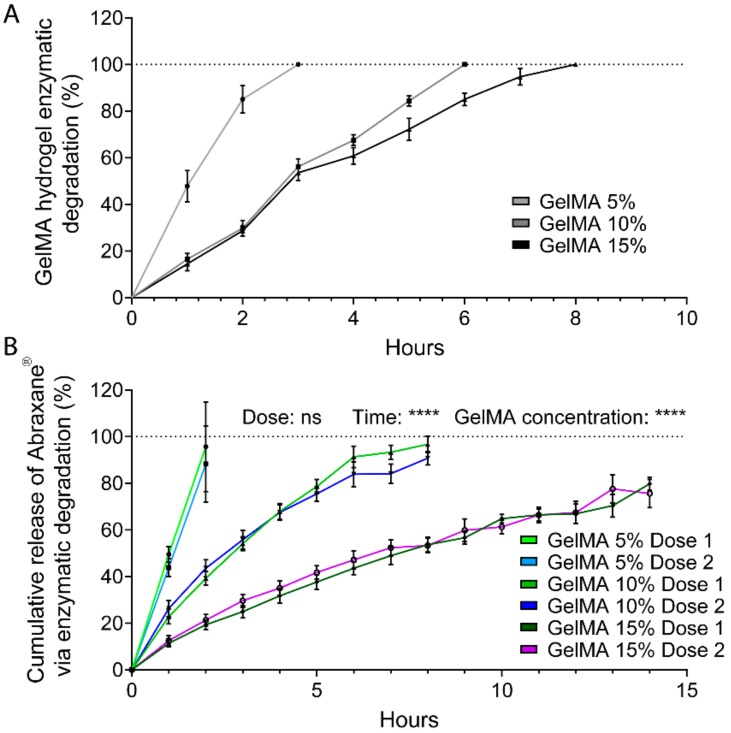
Release kinetics of FITC-labeled Abraxane^®^ from GelMA hydrogels via enzymatic (collagenase II) degradation. (**A**) GelMA degradation over time, as measured by mass loss normalized to initial mass. Data are shown as means ± standard deviation (SD), *n* = 4. (**B**) Cumulative release profiles, normalized to respective free doses suspended in collagenase II. Dose 1 and 2 refer to 37.5 µg and 75 µg Abraxane^®^, respectively. Data are shown as mean ± SD, *n* = 4. **** *p* < 0.0001; *** *p* < 0.001; ** *p* < 0.01; * *p* < 0.05.

GelMA contains natural degradation sites for collagenases and matrix metalloproteinases (MMPs) and undergoes enzymatic degradation once implanted in the body [53,55]. Hence, in a clinical setting, Abraxane^®^ would also be released via proteolytic degradation of GelMA. We, therefore, investigated the in vitro degradation of 5%, 10%, and 15% GelMA-DDS formulations and effects on the release of FITC-labeled Abraxane^®^ (Figure 4). As expected, the time to full degradation increased with GelMA concentration. The 5% GelMA hydrogels degraded within 3 h, while 10% and 15% GelMA followed a similar degradation rate for the first four hours and completely degraded after 6 h and 8 h, respectively. The linearity of the hydrogel degradation rate suggests a near zero-order degradation kinetic which occurs when the rate of hydrogel bond cleavage is faster than the diffusion rate of the enzyme solution through the hydrogel and is characteristic of a surface erosion [50,51,70,71]. Enzymatic release of the drug followed a similar trend; however, degradation was slower compared to GelMA alone, and the GelMA-DDS degraded within 2 h, 8 h, and 14 h for 5%, 10%, and 15% GelMA, respectively, irrespective of the Abraxane^®^ dose. For both doses and all GelMA concentrations, enzymatic degradation led to a complete release of the encapsulated drug, suggesting that a combination of diffusion and proteolytic degradation may improve the drug delivery efficiency *in vivo*.

### 3.6. Aggregation of Abraxane^®^

**Figure 5:**
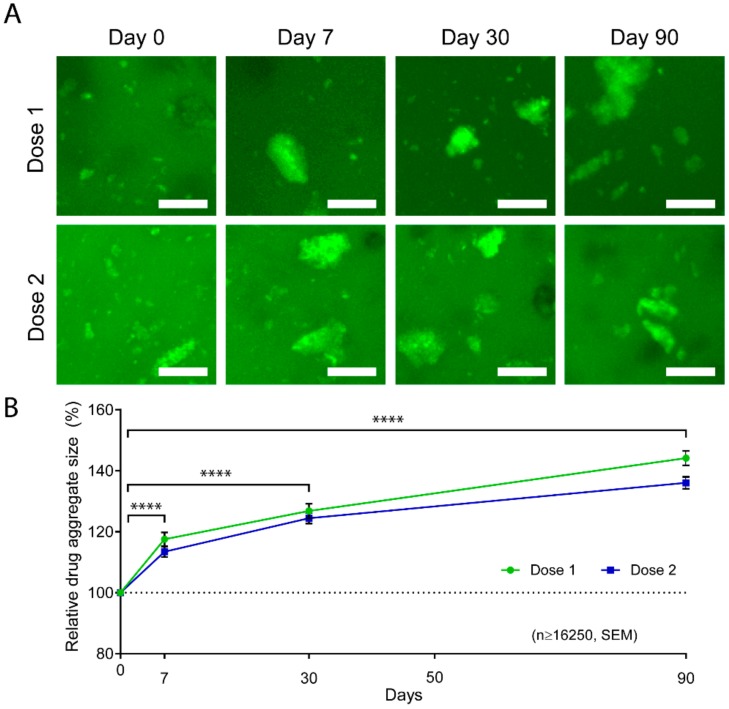
Aggregation of FITC-labeled Abraxane^®^ in GelMA hydrogels. (**A**) Representative *z*-stack maximum projections of 10% GelMA releasing FITC-Abraxane^®^ for Dose 1 and 2 formulations. Scale bar = 25 µm. (**B**) Relative average aggregate size normalized to Day 0 and shown as ± standard error of the mean, *n* ≥ 16,250 aggregates, **** *p* < 0.0001.

Our previous in vitro Abraxane^®^ release data indicated that the release mechanism was mainly diffusion-driven and achieved a maximum cumulative release of 67% of Dose 1 and 75% of Dose 2 in 5% GelMA. This data suggests that part of the drug was larger than the hydrogel mesh size and thus retained in the hydrogel. In fact, albumin has been largely used as a model protein, and its tendency to aggregate via electrostatic interactions has been previously reported and investigated for the application of protein release [38,72,73,74,75]. We, therefore, hypothesized that partial aggregation of Abraxane^®^ was occurring in the GelMA-DDS. Consequently, smaller drug particles were diffusing first during the first hours of release because since they were small enough, whereas bigger drug aggregates were either slowed down or entrapped in the hydrogel. To investigate this, we used Doses 1 and 2 formulations in 10% GelMA and imaged the FITC-labeled Abraxane^®^ at different timepoints of the in vitro release. We were able to confirm partial aggregation of Abraxane^®,^ as evidenced by the presence of large particles in the GelMA-DDS. We found an increase in the aggregate size over time for both Dose 1 and Dose 2 formulations (Figure 5A). The average aggregate size increased significantly throughout the release assay to reach 144.15% and 136.06% of the initial sizes on Day 0 for Dose 1 and Dose 2 formulations, respectively (Figure 5B). The size distribution of the aggregates confirmed the reduction of the smallest particle fraction, i.e., bin size 0 to 500 µm^3^, from Day 0 to Day 90 for both formulations, whereas the relative frequencies of larger aggregates increased (Figure S5). In other words, the average drug particle size increased throughout the release assay because of smaller particles being released from the GelMA-DDS first, confirming the hypothesis.

### 3.7. In Vitro Evaluation of the GelMA-DDS

Last, we investigated the cytotoxic effect of the proposed GelMA-DDS on triple-negative MDA-MB-231 breast cancer cells in 3D culture, using Dose 1 and Dose 2 formulations in 10% GelMA. This hydrogel concentration was chosen because it achieved a more sustained release of the drug compared to 5% GelMA and had a higher encapsulation efficiency than 15% GelMA. The GelMA-DDS was compared to the controls containing the same Abraxane^®^ free doses in cell culture media.

**Figure 6:**
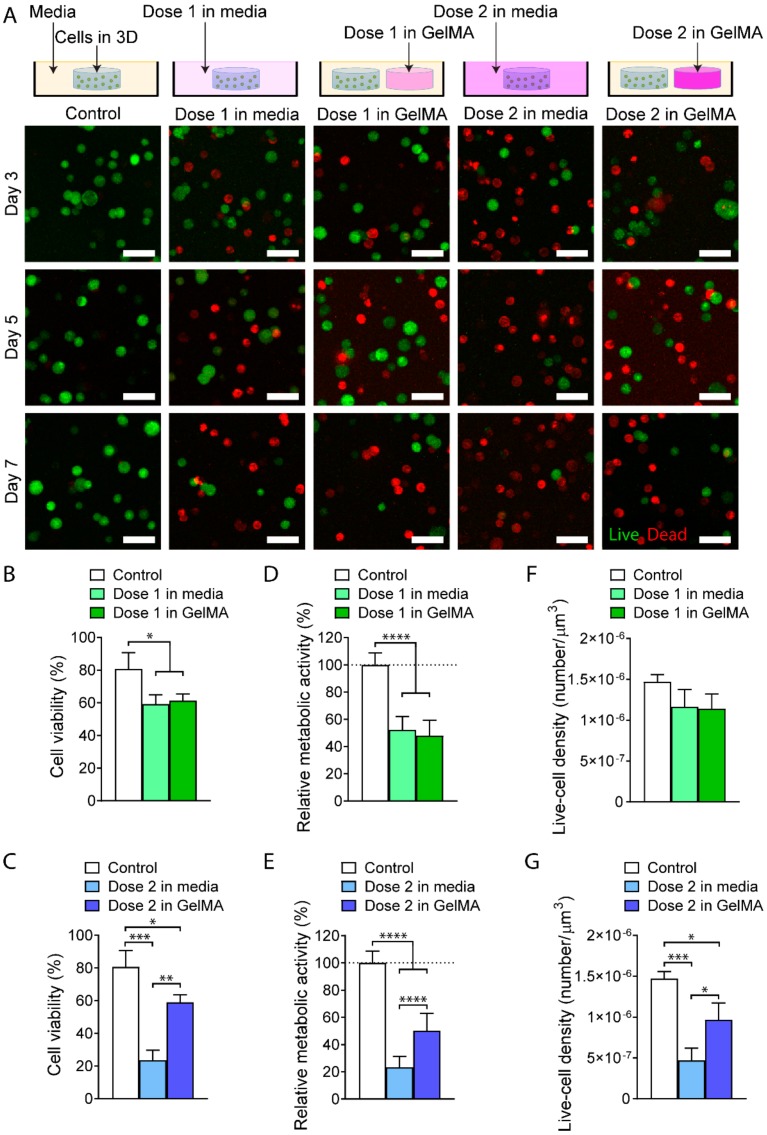
In vitro evaluation of GelMA-DDS 10% on human MDA-MB-231 breast cancer cells cultured in separate three-dimensional (3D) GelMA hydrogels. (**A**) Maximum projections of z stacks with live/dead staining using Fluorescein Diacetate (FDA) and Propidium Iodide (PI) of cancer cells for five media conditions; no drug (control), Dose 1 free in solution, Dose 1 in GelMA, Dose 2 free in solution, and Dose 2 in GelMA (from left to right). Green represents live cells, and red represents dead cells. Scale bar = 50 µm. GelMA (**B**–**G**) Quantitative in vitro assessment of GelMA-DDS formulations at Day 7; viability (**B**–**C**), metabolic activity (**D**–**E**), and Live-cell density (**F**–**G**) for no drug control, drug in media and drug in GelMA. Data are shown as mean ± standard deviation. The experiment was repeated three times, n=3. **** *p*<0.0001; *** *p*<0.001; **p<0.01; * *p*<0.05.

Live/dead staining of MDA-MB-231 cells (Figure 6A) revealed increased cell death in the drug-treated groups compared to the control. By Day 7, both Dose 1 in media and Dose 1 in GelMA-DDS decreased cell viability significantly to approximately 60% (Figure 6B). Similarly, Dose 2 from GelMA-DDS significantly reduced cell viability to around 60%. However, Dose 2 in media caused a significantly higher decrease in cell viability, reaching 23.5% (Figure 6C). The same trend was observed for the metabolic activity of the cells (Figure 6D,E). A significant decrease of 50% was observed for Dose 1 in media, Dose 1 in GelMA-DDS, and Dose 2 in GelMA-DDS, whereas the free Dose 2 group achieved a reduction to 23.3% relative metabolic activity, which was significantly lower than Dose 2 in GelMA-DDS. Regarding the live cell density (Figure 6F,G), a drop to 80% was observed for both Dose 1 in media and Dose 1 in GelMA-DDS groups. The Dose 2 GelMA-DDS formulation significantly reduced the live cell density to 65%, and the ‘Dose 2 in media’ group significantly reduced it further down to 32%. Looking at the three chosen indicators, i.e., cell viability, metabolic activity, and live-cell density, Dose 1 of Abraxane^®^ delivered by 10% GelMA-DDS matched the performance of the same dose suspended in the media. On the contrary, the bioactivity of Dose 2 released from GelMA seemed to have been impaired as it was not as efficient as the same dose in media. Furthermore, the action of Dose 2 in GelMA-DDS was limited and equaled cytotoxic effect and metabolic activity reduction of Dose 1. This event seemed to be driven by the dose parameter but also protein interactions when encapsulated in GelMA, which could be explained by the aggregation of Abraxane^®^ at higher concentrations. Drug aggregation in the hydrogel appears to have reduced and limited the availability of Paclitaxel to the cancer cells; hence, we have demonstrated that overall, there may be reduced encapsulation and release efficiency at higher drug loading, and thus that lower drug loadings may be preferred.

## 4. Conclusions

We successfully encapsulated Abraxane^®^ in GelMA hydrogels with high encapsulation efficiencies. Mechanical characteristics of the drug delivery system were highly tunable and proved to be unaffected by drug loading. The in vitro release was characterized by a larger release for the first hour, followed by a slower release of Abraxane^®^. The initial release was affected by both the dose and the GelMA concentration: it increased with the higher dose and was diminished by the increase of macromere content. Overall, Abraxane^®^ release kinetics were strongly dependent on GelMA concentration, and increased release sustainability was achieved with increasing concentrations. Since the GelMA concentration of 15% displayed a lower loading yield, we suggest limiting the GelMA concentration at 10%. Confocal microscopy revealed partial aggregation of the FITC labeled drug in the hydrogel and an increase in average aggregate size over time. Enzymatic degradation of the GelMA-DDS enabled the complete release of the drug cargo. Finally, Abraxane^®^ delivery significantly reduced the viability, metabolic activity, and live-cell density of 3D MDA-MB-231 cells *in vitro*. Consequently, our GelMA-DDS, which provides a controlled and localized release of Abraxane^®^, could be used to treat and prevent cancer recurrence at the primary site after tumor resection. This fundamental study is the necessary foundation for the development and characterization of the GelMA-DDS presented here and thus focused on the in vitro mechanisms and system characterizations. Further studies will be necessary to delineate the behavior and efficacy of the new system using in vivo models of the disease. Ultimately, the ability of GelMA-DDS to be used as a simple injectable hydrogel opens the door to further tailoring for local injections after tumor resection, with drug amounts and hydrogel volumes tuned to the need of each patient and specific chemotherapeutic regimen.

## Figures and Tables

**Table 1:** Fluorescein-5-isothiocyanate (FITC)-Abraxane^®^ encapsulation efficiency in gelatin methacryloyl (GelMA). Results are normalized to control (same amount of free drugs in solution), shown as means and standard deviations, *n* = 4.

Groups	GelMA 5% Dose 1	GelMA 5% Dose 2	GelMA 10% Dose 1	GelMA 10% Dose 2	GelMA 15% Dose 1	GelMA 15% Dose 2
Encapsulation efficiency (%)	95.67	88.29	96.73	90.73	79.80	75.61
Standard deviation (%)	19.21	16.38	3.42	2.88	2.73	6.06

**Table 2:** Korsmeyer–Peppas model (log of % Abraxane^®^ released vs. log time). For each condition, R^2^ values were determined, and the *n* value was used as an indication of the drug release mechanism. The model is valid and was applied only for the first 60% of release.

Condition	Korsmeyer-Peppas Exponent (*n*)	R^2^
Dose 1, GelMA 5%	0.1935	0.9822
Dose 1, GelMA 10%	0.2018	0.9575
Dose 1, GelMA 15%	0.2157	0.9237
Dose 2, GelMA 5%	0.1842	0.9844
Dose 2, GelMA 10%	0.2137	0.9942
Dose 2, GelMA 15%	0.2397	0.9514

## Supplementary Materials

The following are available online at https://www.mdpi.com/2073-4360/12/2/501/s1, Figure S1. MDA-MB-231 and MCF-7 response to drug treatment in two dimensions (2D)/3D. Materials and Method for Figure S1. Figure S2. Effect of UV exposure on fluorescence intensity of FITC-labeled Abraxane^®^. Figure S3. Characterization of release for GelMA-DDS formulations, done in PBS pH 7.4, at 75 rpm agitation and 37 °C. Figure S4. FITC-labeled Abraxane^®^ released per timepoint. Figure S5. The size distribution of FITC-labeled Abraxane^®^ aggregates at the beginning and the end of the release from GelMA-DDS.
