# Supplementary material for: Gelatin Methacryloyl Hydrogels Control the Localized Delivery of Albumin-Bound Paclitaxel
Source: Polymers (Basel). 2020 Feb 24;12(2):501. doi: 10.3390/polym12020501 (PMC7077643; doi:10.3390/polym12020501)
Supplement: Supplementary file 1 [file polymers-12-00501-s001.pdf]

Supplementary Material: Gelatin methacryloyl hydrogels control the localized delivery of albumin-bound Paclitaxel

Margaux Vigata [1,4], Christoph Meinert [1,4,*], Stephen Pahoff [1,4], Nathalie Bock[1,2,5,*] and Dietmar W. Hutmacher [1,3,4,5,*]

[1] Institute of Health and Biomedical Innovation (IHBI), Queensland University of Technology (QUT), Kelvin Grove, QLD, 4059, Australia.
[2] Translational Research Institute, Woolloongabba QLD 4102, Australia.
[3] Australian Research Council Industrial Transformation Training Centre in Additive Biomanufacturing, QUT, Brisbane, Queensland 4059, Australia.
[4] School of Chemistry, Physics and Mechanical Engineering, Science and Engineering Faculty (SEF), QUT, Brisbane, QLD, Australia.
[5] School of Biomedical Sciences, Faculty of Health, QUT, Brisbane, QLD, Australia.
[*] Correspondence: Institute of Health and Biomedical Innovation, Queensland University of Technology, 60 Musk Avenue, Kelvin Grove 4059, QLD, Australia. Emails: christoph.meinert@qut.edu.au, n.bock@qut.edu.au, dietmar.hutmacher@qut.edu.au.

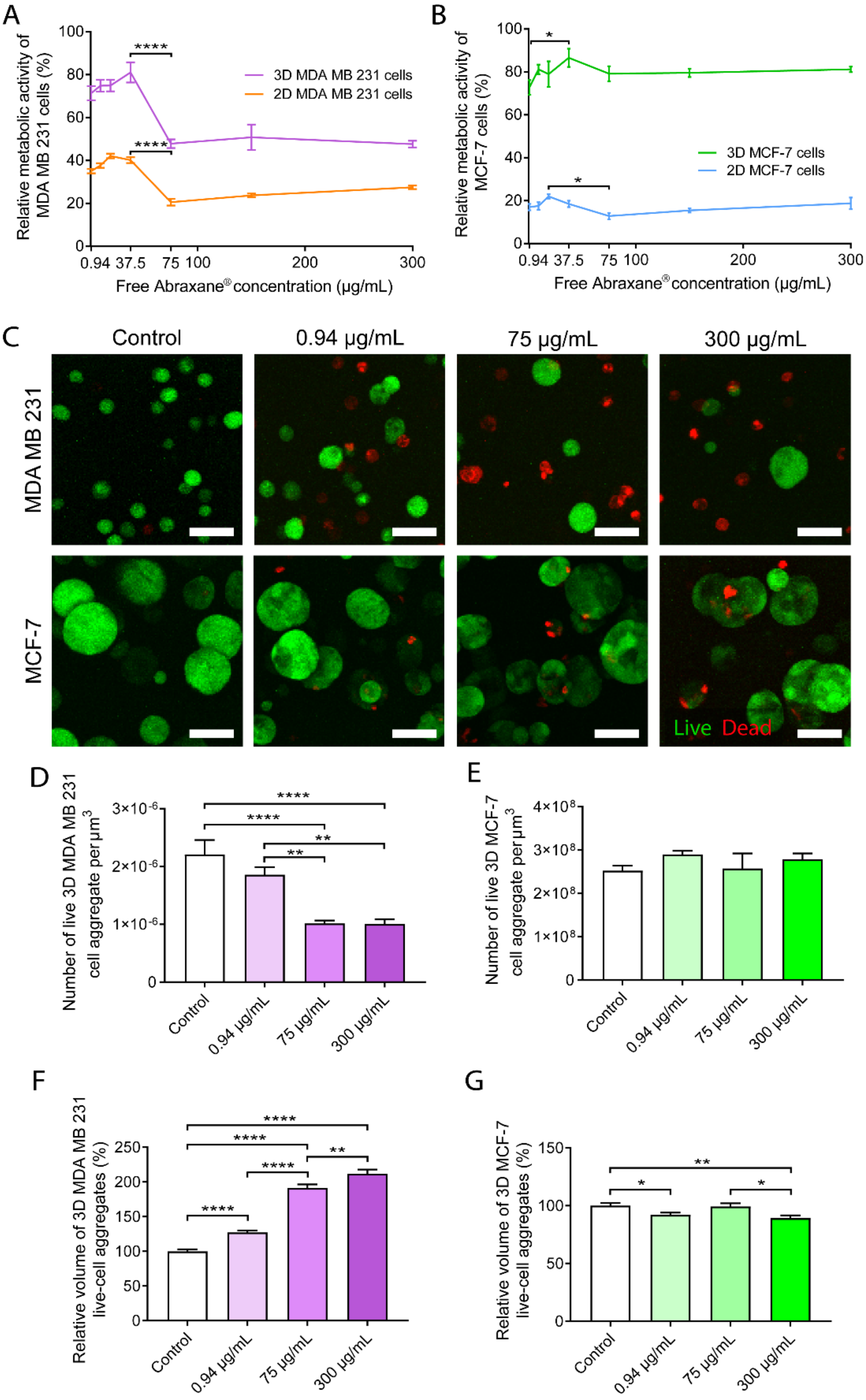

Figure S1. MDA-MB-231 and MCF-7 response to drug treatment in 2D/3D. Cultures were exposed to Abraxane® for 3 days and analysis performed on day 3. A-B) AlamarBlue® analysis on day 3 of 2D and 3D MDA MB 231 cells (A) and 2D and 3D MCF-7 cells (B). Data are shown as means ± standard error of the mean (SEM), normalized to the untreated control group (0 µg/mL = 100 %). C) Maximum projections of *z*-stacks with live/dead staining with Fluorescein Diacetate (FDA) and Propidium Iodide (PI) of 3D MBA MB 231 and 3D MCF-7 cells in media without any drug (control), in media with 0.94 µg/mL, 75 µg/mL and 300 µg/mL of Abraxane®. Scale bar = 50 µm. D-E) Number of live MDA-MB-231 (D) and MCF-7 (E) cell aggregates per µm³ of hydrogel. F-G) Volume of MDA-MB-231 (F) and MCF-7 (G) live-cell aggregates; results are presented as a percentage of the control. Data are

shown as mean ± SEM. 2D experiments were repeated 3 times, n=4 and 3D experiments were repeated twice, n=3. **** $p<0.0001$;*** $p<0.001$; ** $p<0.01$; * $p<0.05$.

Materials and Method for Figure S1

Maintenance

MDA-MB-231 cells were cultured in adherent monolayers in T75 flasks using Dulbecco's Modified Eagle's Medium (DMEM, 10313-021, Thermo Fischer Scientific, USA), containing 10% Fetal Bovine Serum (FBS, Hyclone, Logan, UT, USA) and 1% penicillin/streptomycin (P/S). MCF-7 cells were cultured similarly, only using RPMI 1640 media (11835-030, Thermo Fischer Scientific, USA) supplemented with 10% FBS and 1% P/S, 1X MEM non-essential amino acids (Life Technologies), 1 mM Sodium Pyruvate (Sigma Aldrich, Munich, Germany) and 0.1% human insulin (Life Technologies). Cells were incubated at 37°C and 5% CO_2. Media was changed every 3 to 4 days, and cells were passaged upon reaching confluence.

3D cell culture

The cells were trypsinized, counted and suspended at 1×10^6 cells/mL in a solution of 10% w/v GelMA containing 0.5 mg/mL Irgacure2959. The mixture was transferred into Teflon molds to form disc-shaped hydrogels of 5 mm diameter and 1.8 mm height. UV crosslinking at 365 nm light at an intensity of ~2.6 mW/cm2 in a CL-1000 crosslinker (UVP, Upland, CA, USA) 365 nm was applied for 10 minutes. The gels were then placed in 24-well plates with 1 mL of appropriate culture media, which was changed every 3 to 4 days. Gels were left in culture for 8 days to allow for cell proliferation and aggregate/spheroid formation.

2D dose range study

Cells were trypsinized, counted then seeded in 48 well plates at 8,000 cells/well = 7273 cells/cm^2 for MCF-7 cells and 16,000 cells/well = 14,545 cells/cm^2 for the MDA-MB-231 cells and left incubating overnight at 37°C and 5% CO_2 in a humidified cell culture incubator. On Day 0, drug treatment was applied, i.e., media was changed and replaced by 0.5 mL of new media containing no drug for the control group and the free drug for the drug-treated groups (0.94 µg/mL, 9.4 µg/mL, 18.75 µg/mL, 37.5 µg/mL, 75 µg/mL, 150 µg/mL, 300 µg/mL). On Day 3, metabolic activity was assessed using the AlamarBlue® assay. Cells were incubated for 4 hours with 250 µL of a 10% AlamarBlue® solution v/v (in the appropriate media) at 37°C and 5% CO_2. The fluorescent emission at 590 nm (excitation 544 nm) was measured in a fluorescence plate reader (BMG PolarStar). The experiment was repeated 3 times; n=4 and results were normalized to the control group.

3D dose range study

The protocol was the same as with 2D cells: drug treatment was applied on Day 0; media was changed and replaced by fresh media with the drug concentrations or no drug for the control group only (1 mL per well, 24 well plate). On Day 3, metabolic activity was assessed using AlamarBlue® assay (500 µL of a 10% AlamarBlue® solution v/v in the appropriate media and 4 hours incubation time); and live/dead staining was performed using Fluorescein Diacetate and Propidium Iodide (FDA/PI) for confocal imaging of control, 0.94 µg/mL, 75 µg/mL and 300 µg/mL groups. The Imaris software (Bitplane, South Windsor, CT) was used to reconstruct into 4D animations the live/dead z stacks and determine live cell number and volume. The experiment was repeated 2 times, n = 3, results were reported as a percentage of the control group.

Statistical analysis

The significant difference in means between groups was determined using one-way ANOVA with Tukey post hoc using GraphPad Prism software (version 8, GraphPad, CA, USA).

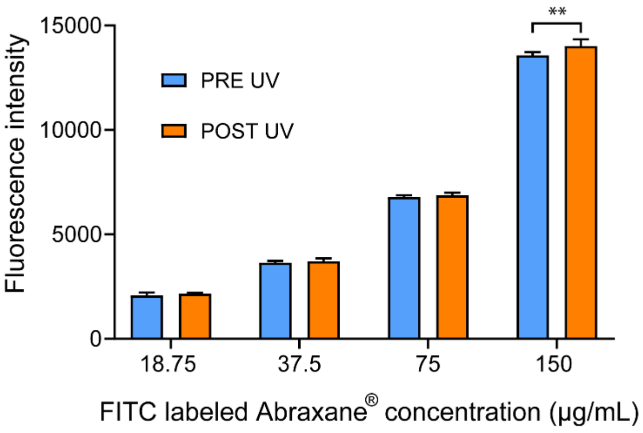

Figure S2. Effect of UV exposure on fluorescence intensity of FITC-labeled Abraxane®. Four concentrations (18.75 μg/mL, 37.5 μg/mL, 75 μg/mL and 150 μg/mL) of FITC-Abraxane were prepared in PBS and dispensed into two 96 well plates. One well plate underwent UV exposure for 30 minutes. Next, Fluorescence intensity was detected using a fluorescence plate reader (BMG PolarStar) at 485 nm excitation and 520 emission wavelengths. Results shown as means ± standard deviation. The significant difference in means between groups was determined using one-way ANOVA with Tukey post hoc using GraphPad Prism software (version 8, GraphPad, CA, USA). ** *p*<0.01.

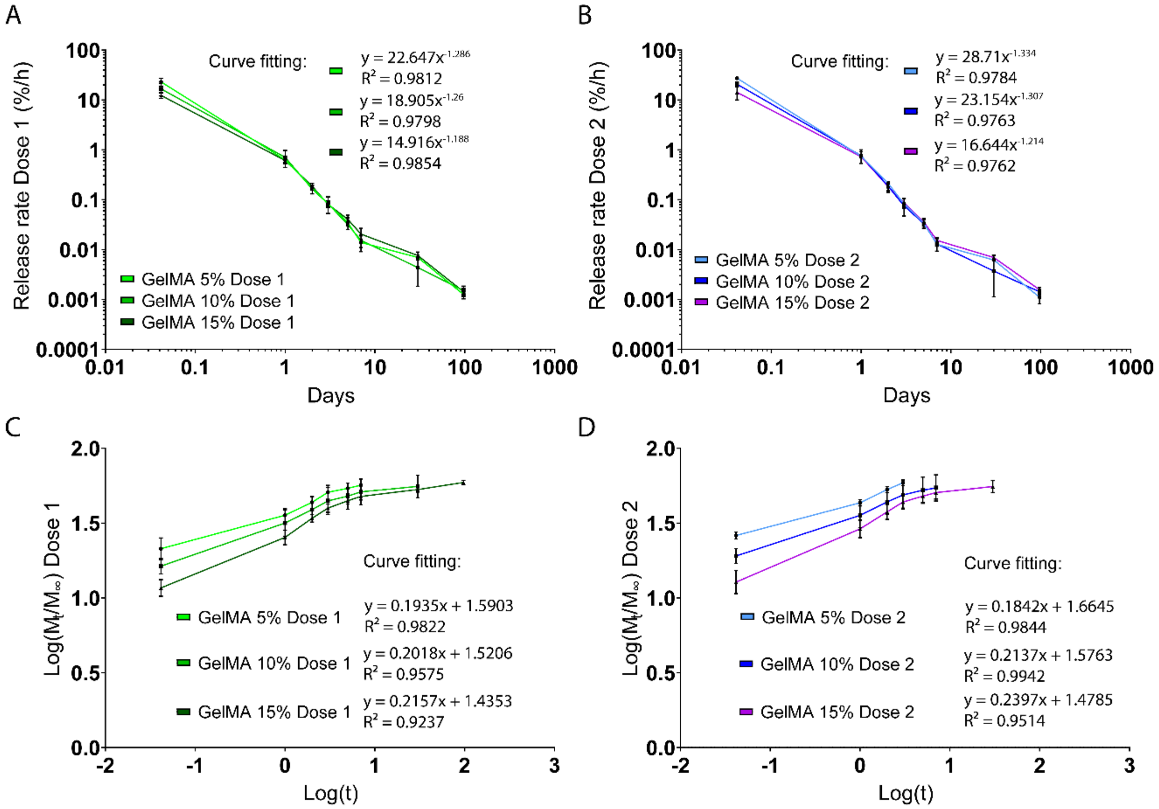

Figure S3. Characterization of release for GelMA-DDS formulations, done in PBS pH 7.4, at 75 rpm agitation and 37 °C. A-B) Release rates from 5%, 10% and 15% w/v GelMA for Dose 1 (A) and Dose 2 (B) formulations. C-D) Korsmeyer – Peppas model for the mechanism of drug release (first 60% drug release) in 5%, 10%, and 15% w/v GelMA for Dose 1 (A) and Dose 2 (B) formulations. Data are shown as mean ± standard deviation. Release experiments were repeated 3 times, n ≥ 5.

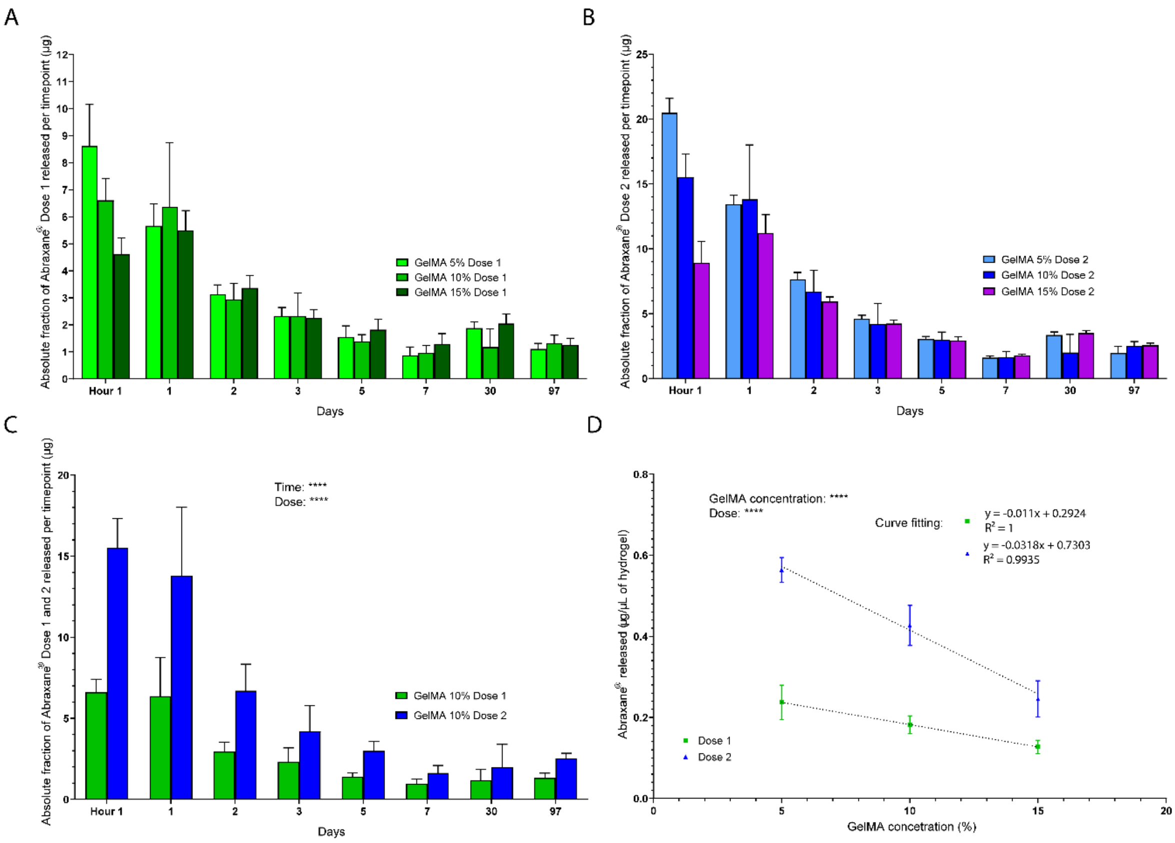

Figure S4. FITC-labeled Abraxane® released per timepoint. A-B) Absolute release values of FITC-labeled Abraxane® over time for various GelMA-DDS formulations (5, 10, 15% GelMA) for Dose 1 (A) and Dose 2 (B) formulations. C) Absolute release values of FITC-labelled Abraxane® over time for GelMA-DDS 10% for Dose 1 and 2. D) Release values of FITC-labelled Abraxane® normalized to GelMA volume after 1 hour of release, for GelMA-DDS formulations of increasing GelMA concentrations. Dose 1 and 2 refer to a loading of 37.5 µg and 75 µg of Abraxane®, respectively. Curve fitting shows an inverse linear correlation between the amount released and the GelMA concentration.

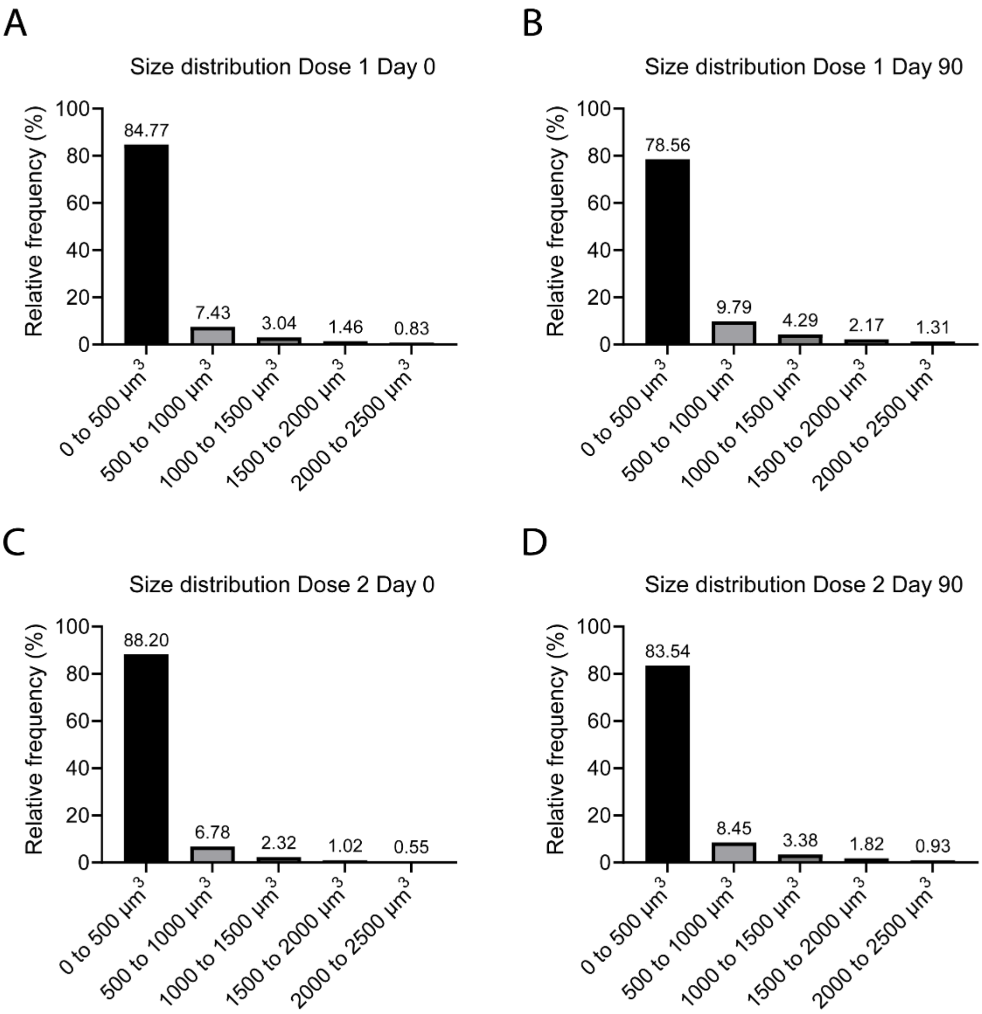

Figure S5. Size distribution of FITC-labeled Abraxane® aggregates at the beginning and the end of the release from GelMA-DDS. A) Day 0 for Dose 1. B) Day 90 for Dose 1. C) Day 0 for Dose 2. D) Day 90 for Dose 2. Bin size = 500 μm³. Dose 1 and 2 refer to a loading of 37.5 μg and 75 μg of Abraxane®, respectively. n ≥ 19517.
